# Stress in nursing workers caring for people with COVID-19

**DOI:** 10.1590/0034-7167-2023-0542

**Published:** 2024-12-13

**Authors:** Carla Barbosa de Menezes, Maria Lúcia Silva Servo

**Affiliations:** I Universidade Estadual de Feira de Santana. Feira de Santana, Bahia, Brazil

**Keywords:** Occupational Stress, Occupational Health Policy, Mental Health, Nurses, COVID-19, Estrés Laboral, Política de Salud Ocupacional, Salud Mental, Enfermeros, COVID-19

## Abstract

**Objectives:**

to analyze stress from the perspective of nursing workers caring for people with COVID-19 in a public hospital in the Recôncavo region of Bahia.

**Methods:**

this is an exploratory qualitative study, conducted through semi-structured interviews. The data were analyzed using word clouds, similarity trees, and content analysis.

**Results:**

nursing workers were exposed to stress while attending to patients with COVID-19. The reported stressors in the workplace included: work overload, lack of planning, speed in performing tasks, fatigue, lack of participation in decision-making, lack of support from management, technological changes, excessive responsibility without preparation, interpersonal conflicts, and professional undervaluation.

**Conclusions:**

exposure to these stressors leads to emotional exhaustion and demotivation, which were intensified during the COVID-19 pandemic.

## RESUMEN

**Objetivos:**

analizar el estrés desde la perspectiva de los trabajadores de Enfermería en la atención a las personas con COVID-19 en un hospital público del recôncavo baiano.

**Métodos:**

se trata de una investigación cualitativa de carácter exploratorio, realizada a través de entrevistas semiestructuradas. Los datos fueron analizados mediante nubes de palabras, árbol de similitud y análisis de contenido.

**Resultados:**

los trabajadores de Enfermería estuvieron expuestos al estrés al atender a pacientes con COVID-19. Los estresores reportados en el ambiente laboral fueron: sobrecarga de trabajo, falta de planificación, rapidez en la realización de tareas, fatiga, falta de participación en la toma de decisiones, falta de apoyo por parte de la gestión, cambios tecnológicos, exceso de responsabilidad sin preparación, conflictos interpersonales y desvalorización profesional.

**Conclusiones:**

el contacto con estos estresores conlleva desgaste emocional y desmotivación, intensificados durante la pandemia de COVID-19.

## INTRODUCTION

Stress among healthcare workers-particularly nursing professionals-is a topic that fosters many debates and investigations. Nursing is a category exposed to high levels of pressure and stress, which can trigger mental disorders characterized by symptoms such as anxiety, memory and concentration difficulties, fatigue, insomnia, and stress^(1)^.

Occupational stress differs from common stress in that work is the essential factor for its development; this type of stress occurs when the worker cannot act on the causative agents, breaking the adaptation, with stress symptoms persisting and the organism becoming exhausted^(2)^.

It is known that the hospital is a stress-generating environment for workers, especially nurses, who face multiple demands, such as dealing with pain, suffering, death, and loss, and requiring great skill in handling service users, among others-which consequently makes workers more vulnerable to depressive feelings and emotional exhaustion. Adding to these factors are interpersonal relationships, unfavorable working conditions, and low remuneration, all of which contribute to the development of stress^(3)^.

Based on the World Health Organization (WHO) report, disclosed by the Federal Nursing Council (COFEN, in Portuguese)^(4)^, stress factors related to nursing work tend to exacerbate in a global public calamity scenario, such as the COVID-19 pandemic that occurred in 2020, 2021, and 2022. Unfortunately, 19,026 nursing workers were confirmed with COVID-19, among whom 833 died.

Studies have shown that the high number of deaths and long working hours without adequate structure due to COVID-19 among nursing workers and other health professionals generated physical and emotional stress, depression, and insomnia^(5-7)^. Given this, it is important to develop works that provide for the creation of interventions and protective measures for nursing workers.

## OBJECTIVES

To analyze the stress experienced by nursing workers caring for people with COVID-19 in a public hospital in the Recôncavo region of Bahia.

## METHODS

### Ethical Aspects

The project was submitted to and approved by the Human Research Ethics Committee of the State University of Feira de Santana (UEFS). The Informed Consent Form (ICF) was signed by the participants after being informed about the objectives, risks, and benefits of the research. Participants were identified by the initials Interviewee (INT) and the corresponding interview numbers. Participants were guaranteed the right to decline to answer any questions without explanation or justification and could withdraw from the study at any time without any repercussions.

### Type of Study

This is a descriptive qualitative study conducted through semi-structured interviews, following a script and constructed in accordance with the Consolidated Criteria for Reporting Qualitative Research (COREQ)^(8)^. This manuscript is part of a master’s dissertation presented to the Graduate Program in Public Health at UEFS.

### Study Setting

The study was conducted at Hospital Municipal Nossa Senhora da Natividade, located in the municipality of Santo Amaro, in the state of Bahia. The interviewees are nursing workers who provided care to people with COVID-19 while fully engaged in their professional duties and listed in the National Registry of Health Facilities (CNES in Portuguese). The institution provides emergency and urgent care; urology, cardiology, ambulatory blood pressure monitoring, and electrocardiogram services; neurology with electroencephalogram, among others. All services are provided to meet the demand of the local and neighboring municipalities.

The hospital serves an average of 250 patients per day, with 35 beds for emergency, urgent care, adult and pediatric care, medical clinic, and pediatric inpatient services. During the pandemic, the hospital was used to care for patients diagnosed with COVID-19 for a period of three years, offering 15 beds dedicated to assisting these patients.

### Data Source

To access the study participants, authorization was requested from the Municipal Health Department of Santo Amaro for the research, as well as contact information such as phone numbers and emails of the workers affiliated with the unit.

Initially, 20 participants, including nurses and nursing technicians, were approached for the interview. Among them, three declined, citing shyness, and one expressed a lack of interest. Thus, a total of 16 participants were interviewed, with audio recordings made using a cellphone for transcription purposes.

The exclusion criteria established were: nursing workers on medical leave or maternity leave, on vacation, and/or those who did not wish to participate in the study. In Chart 1, all the participants in the study were characterized and described.

**Chart 1:** Characterization of nursing workers who provided care to people with COVID-19 at Hospital do Recôncavo Baiano in Santo Amaro, Bahia, Brazil, 2023

No	Age	Gender	Professional Training	Time Since Training	Time in Profession	Time at Institution	Work Shift	Other Employment
**1**	27	Feminine	Nursing Technician	4 years	4 years	4 years	36 hrs/weekly	No
**2**	29	Feminine	Nursing Technician	4 years	4 years	4 years	36 hrs/weekly	No
**3**	25	Feminine	Nursing Technician	6 years	5 years	2 years	36 hrs/weekly	Yes
**4**	36	Feminine	Nursing Technician	3 years	3 years	2 years	36 hrs/weekly	No
**5**	42	Feminine	Nurse	5 years	5 years	2 years	36 hrs/weekly	Yes
**6**	43	Feminine	Nurse	4 years	4 years	4 years	36 hrs/weekly	Yes
**7**	35	Feminine	Nurse	8 years	4 years	1 year	36 hrs/weekly	No
**8**	35	Feminine	Nurse	13 years	12 years	2 years	36 hrs/weekly	Yes
**9**	54	Masculine	Nurse	4 years	3 years	2 years	36 hrs/weekly	No
**10**	36	Feminine	Nurse	7 years	3 years	3 years	36 hrs/weekly	No
**11**	34	Masculine	Nurse	3 years	2 years	2 years	36 hrs/weekly	No
**12**	36	Feminine	Nurse	3 years	2 years	2 years	36 hrs/weekly	No
**13**	24	Feminine	Nursing Technician	2 years	2 years	2 years	36 hrs/weekly	Yes
**14**	50	Feminine	Nursing Technician	9 years	6 years	3 years	36 hrs/weekly	No
**15**	37	Feminine	Nurse	7 years	7 years	4 years	36 hrs/weekly	Yes
**16**	46	Feminine	Nursing Technician	8 years	8 years	8 years	36 hrs/weekly	No

### Data Collection and Organization

The semi-structured interview was conducted from December 2022 to February 2023, divided into two parts: the first focused on the characterization of the participants, and the second on stress among nursing workers. The interviews were conducted individually by the principal researcher, with prior scheduling of date, time, and place, according to the participant’s availability.

The guiding questions of the interview were:

1. What do you understand by stress?
2. Describe what work was like during the pandemic.
3. Did you experience any stressful situations in your daily care of people with COVID-19? If so, how did you handle these situations?

The interview with each participant lasted around 20 to 30 minutes, and at the end, participants had the opportunity to listen to the recordings to correct their responses if necessary and to consent or not to their participation in the project. The interviews concluded when the participants’ conceptions, explanations, and meanings no longer contributed to theoretical reflection and became repetitive. It should be noted that after the transcriptions were completed, they were sent to the participants for validation.

Once the data collection was completed, the data obtained during the recordings and the ICF were downloaded and sent to the personal email of the responsible researcher. Subsequently, the files were stored on a flash drive so that all data could be deleted from the cellphone.

### Data Analysis

The characterization of the study participants was entered into Microsoft Word^®^. Additionally, the results of the responses related to the central theme of this research were organized using the following tools:

- **Similarity Analysis:** The textual corpus was analyzed using IRAMUTEQ software (Interface de R pour les Analyses Multidimensionnelles de Textes et de Questionnaires), version alpha 2^(9)^. This free software functions as an R interface, suitable for managing and statistically processing texts from interviews and open-ended questionnaires; transcription was done using Apache OpenOffice^®^ to avoid coding errors.
- **Word Cloud:** This tool organizes the interview in such a way that the larger and more centralized a word is in the cloud, the greater its representativeness in the corpus under analysis. Conversely, the farther and smaller a word is, the lesser its degree of evocation^(10)^.
- **Content Analysis:** The data from the semi-structured interviews were analyzed and interpreted based on Bardin’s Content Analysis Method^(11)^. This is a category of conversation analysis methods that allows the deduction of knowledge related to the conditions of message production through systematic and objective content description procedures. Furthermore, this method allows for the examination and analysis of what goes beyond the manifest contents.

## RESULTS

**Figure 1:**
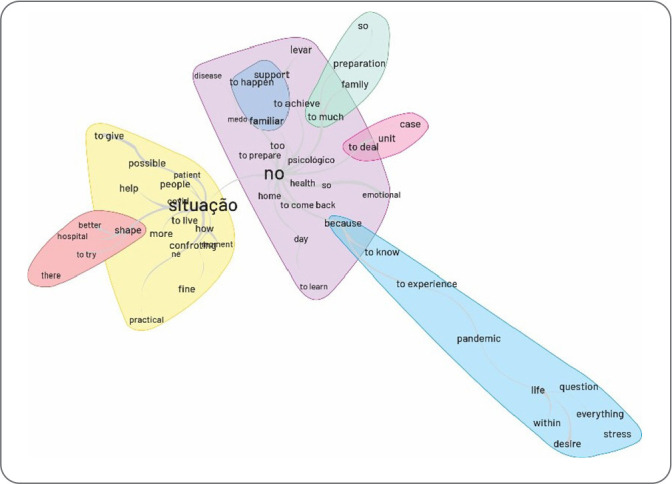
Meaning and experience of stress by hospital nursing workers during the COVID-19 pandemic

**Figure 2:**
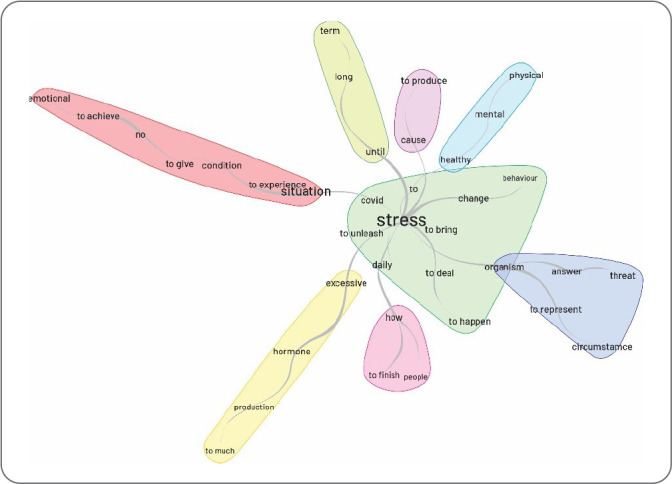
Coping strategies used by hospital nursing workers during the COVID-19 pandemic

From the data obtained, the following categories were identified: the meaning and experience of stress from the perspective of hospital nursing workers who worked during the COVID-19 pandemic (Figure 1); and stress in the daily hospital life of nursing workers caring for people with COVID-19 (Figure 2).

### Category 1 - Meaning and experience of stress by hospital nursing workers during the COVID-19 pandemic

The meaning and experience of stress encompass the understanding that nursing workers have of stress as a conflicting occurrence that affects emotional balance, generating physical and mental reactions in response to the situations they experience:

[...] *Situations that take away our emotional balance due to negative events that happen in our lives* [...]. (Int. two)

[...] *everything that takes us out of our comfort zone* [...] *changes our daily routine, destabilizes our mental health, and causes discomfort in our daily lives* [...]. (Int. five)

It was possible to identify the relationship between the concept of stress and external factors stemming from the work environment, as well as the symptoms caused by excessive stress:

[...] *Stress causes an excessive demand that we often cannot handle, it is when there is excessive pressure and demand; these factors trigger stress* [...]. *Knowing that you are being demanded and pressured* [...]. *Besides dealing with limitations and inadequate conditions to meet the high demand* [...]. (Int. nine)

[...] *Stress is the occurrence of sudden or threatening circumstances, which create a state of alert* [...]. *Situations that cause excitement, irritability, and fear* [...]. (Int. eight)

Regarding the stressors that affect workers’ lives, it was reported that:

[...] *trying to solve problems and address issues that were beyond reach, dealing with the absence of family, managing families wanting access to patients when it was not possible* [...] *the fact that it was new and unknown,* [...] *the lack of information,* [...] *lack of equipment* [...] *a very hostile work environment* [...] *dealing with stressful situations every day in the course of providing care* [...]. (Int. 13)

The workload caused by excessive hours negatively impacts the quality of life, as noted by one of the interviewees:

[...] *work overload, shortage of professionals,* [...] *and insufficient personal protective equipment,* [...] *risk of contamination by COVID-19,* [...] *prolonged use of masks, chronic fatigue, discrimination, physical and verbal violence,* [...] *the exhausting shifts increased the level of stress and dissatisfaction, thus compromising the quality of work service, frustration of not being able to save a life despite all my effort* [...] *the pain of losing without even saying goodbye* [...]. (Int. eight)

### Category 2 - Stress in the Daily Hospital Life of Nursing Workers Caring for People with COVID-19

Nursing workers experienced situations of discrimination and isolation from friends, family, and acquaintances. Due to working on the front lines, they ended up being perceived as a source of contamination for society. As one interviewee confirms:

[...] *I experienced prejudice because we had direct contact with virus carriers and were in the healthcare field; people, including relatives, thought we were transmitters of the disease* [...]. (Int. one)

Regarding stress in the daily lives of nursing workers caring for people with COVID-19 in the hospital, it was reported that:

[...] *there was a fear of contracting the disease and transmitting it to those around us.* (Int. two)

[...] *dealing with the fear of the unknown* [...]. (Int. 14)

Nursing workers understand the heightened fear as a prevalent feeling at that time, especially due to the fear of death, transmission, and contamination amid the chaos. This is evident from the following statement:

[...] *I felt a lot of anxiety, stress, and fear* [...] *fear of dying* [...]. (Int. four)

The COVID-19 pandemic brought significant repercussions on the mental health of nursing workers. This issue was highlighted in the following interview:

[...] *uncertainties* [...] *in addition to the excess information in the media, the politicization of health increased my stress and insecurity more and more, causing a distancing from my family and friends with isolation measures, loss of sleep, irritability, frequent crying, inability to relax, difficulty concentrating, and slow thoughts are the aspects that stood out the most* [...]. (Int. eight)

From the reports, it is noted that there is a sense of insecurity, along with a feeling of helplessness:

[...] *I felt a lot of anxiety, stress, I couldn’t sleep, had insomnia, kept thinking, couldn’t eat, at that time I lost weight, felt very insecure, sad, cried a lot and every time I came home, I cried a lot feeling helpless* [...]. (Int. 12)

[...] *The inability to often provide adequate support to the patient when they needed attention* [...] *the patients’ need for a companion, but the workload made it impossible to try to meet that need, the lack of equipment for the number of patients who needed it, the inability to offer a word of comfort in moments of loneliness, I lived through moments of sadness, seeing the patients’ suffering without a family member nearby, isolated, but the team was giving them attention as much as possible* [...]. (Int. six)

There were negative situations affecting the mental health of workers, demonstrated through various signs and symptoms:

[...] *I started to have insomnia, irritability, frequent crying, inability to relax, and difficulty concentrating, and I lost satisfaction in my career* [...]. (Int. six)

[...] *it was the lack of materials, equipment, a deficit of many things due to the demand; there was a shortage of materials, masks, gloves not only in the city we are talking about now but across the country and the world there was a lack of equipment and materials to provide care to COVID-19 patients* [...]. (Int. five)

In this context, the coping strategies used by nursing workers to deal with stressful situations in their daily hospital life were as follows:

[...] *Calling on God to give us emotional control to act in the best way possible in providing care* [...]. (Int. 14)

[...] *I faced this situation always with a positive mindset, which helped me not to despair or even fall into depression like many other professionals, unfortunately* [...]. (Int. two)

[...] *emotional balance, a true psychological preparation* [...]. (Int. nine)

[...] *I had to control my emotions* [...] *I find it difficult to get psychological help from a distance* [...]. *It didn’t work, I tried, but it didn’t work, so I really had to try to control my emotions* [...]. (Int. 10)

Despite the pandemic period, the opportunity to get the first job was something positive for some of the interviewees:

[...] *immediate hiring, which opened up job opportunities for professionals, especially those who were unemployed and inexperienced, looking for a chance in the job market* [...]. (Int. one)

[...] *the opportunity for new jobs, because this was my first job, and I was looking before COVID-19 emerged* [...]. (Int. 10)

[...] *being newly graduated, it opened up a job opportunity* [...]. (Int. 12)

[...] *job opportunities, especially for those who were unemployed* [...]. (Int. 14)

The participants also reported points regarding the empathetic relationships they experienced during the pandemic period:

[...] *not standing idly by and making a difference in the lives of each person who came into our care, always giving our best. I consoled many families and cried with them for the loss of their loved ones, but I also saw many patients recover and return to their families* [...]. (Int. two)

[...] *the change in perspective on life, on my concept of care, on my concept of humanity, of treatment, and respect for the life of others* [...]. (Int. 13)

[...] *always having a clear conscience that I was doing my best, the sense of accomplishment and being able to help people was gratifying. Another possibility is the personal growth; it was a lesson for all of us, I managed to evolve a lot as a person and as a professional, because I ended up having a different vision, a growth as a human being, as a spirit* [...]. (Int. 15)

In the interview, some positive situations in the daily life of the individual were also highlighted. These situations led to changes in the daily routine and became habits in the worker’s life:

[...] *a great legacy that science advanced, new technologies were discovered, equipment that was previously used on a smaller scale is now being used more, such as the oximeter. Regarding hygiene, it was something that the population developed a habit of washing their hands constantly, maintaining better personal hygiene more regularly. The perspective for this moment was that people evolved in terms of hygiene and self-care* [...]. (Int. two)

## DISCUSSION

The pandemic period was significant for analyzing the stress experienced by nursing workers, as there was an exacerbated fear of dealing with the imminence of death, fear of contamination, and transmission of the disease-especially to family members. As a result, nursing workers highlighted the development of feelings of uncertainty, insecurity, helplessness, irritability, and insomnia. In response to these factors, coping strategies included deepening their religious faith and seeking psychological support through personal resources without institutional help. Positively, there was an increase in the demand for nursing workers and the strengthening of empathetic relationships, leading to significant changes in the work environment.

The participants in this research were mostly female^(12)^, aged between 24 and 54 years, consistent with the findings of Appel, Carvalho, and Santos^(13)^, where the nursing staff was predominantly composed of women (88.5%) aged between 23 and 54 years, with greater vulnerability to developing stress, anxiety, and depression. The prevalence of stress symptoms in women can be attributed to the overload caused by the combination of formal and domestic work, uncertainty regarding socioeconomic-emotional issues, risk of unemployment, home office activities that had to be balanced with family and professional activities, increased occurrences of domestic violence during this isolation period, and the fear of infecting their families with COVID-19^(14-16)^.

It was observed that the participants had been working at this health facility for two to four years, and a total of eight participants started their service during the pandemic period. It is known that the less experience, the greater the predisposition to work-related stress due to the greater difficulty in dealing with encountered stressors, leading to a shortage of nursing workers. This condition aligns with the research, as the workers had a short professional tenure. More experienced professionals tend to have better coping mechanisms for stressful situations^(14)^.

The nursing workers interviewed in the hospital network had an average workload of 36 hours per week, with both day and night shifts, and the vast majority had 12 or more years of professional experience. It is known that the longer the exposure to circumstances that cause physical and cognitive wear and tear in humans, the greater the vulnerability to the worker’s psychological health^(16)^.

Nursing represents the largest health workforce in the world and was the class that exhibited the most fear of contracting the virus due to the risk of contagion. This was the category that experienced the highest rates of infection and mortality during the COVID-19 pandemic^(17)^. Given this premise, the word “stress” in the similarity analysis was the most central, interlinked with the words “experience” and “excessive”, suggesting a relationship between stress and situations that excessively affect mental health.

The word “situation” is accompanied by the words “COVID-19”, “daily life”, and “change”, demonstrating that the changes experienced by nursing workers in their daily hospital routine during the COVID-19 period affected mental health and triggered stress. Work-related experiences during the COVID-19 period were detrimental, including the particularities of work environment organization, workload caused by excessive hours, constant and elevated psychic demands, routine performance issues, a scarcity of workers, and alternating shifts^(18)^. Thus, the scenario brought about by the COVID-19 pandemic introduced numerous changes to the hospital routine, resulting in physical and psychological wear and tear on these workers, consequently triggering stress.

The constant stressful situations experienced by nursing workers were intensified during the COVID-19 pandemic, as they had to deal with death, a shortage of PPE, and high numbers of patients with high viral transmissibility^(19)^. Additionally, there was a need to develop precise and cautious care, both in technical procedures and self-care, to minimize the risks of contamination. These factors led to physical and mental illness, potentially causing nursing workers to be unable to perform their job tasks^(20)^.

Fear is necessary for survival as it involves biological processes that contribute to the organism’s defense in response to threatening events. When exacerbated, as reported by the participants in this study, it becomes harmful, contributing to the development of stress and other psychiatric disorders such as nervousness, inability to relax, insomnia, daytime sleepiness, uncertainty, and insecurity in the face of media information regarding treatment protocols. Coupled with the uncertainty and unpredictability of the COVID-19 pandemic period, this triggered a predisposition for the emergence of stress among nursing workers^(21-24)^. Uncertainty negatively influences the behavior and overall well-being of nursing workers and consequently interferes with the maintenance of the quality of health care provided to the population^(25)^. Furthermore, the feeling of helplessness arises when these workers are unable to change the critical situation of the patient^(18,26)^.

### Coping strategies used by nursing workers

In the similarity tree regarding the coping strategies used by nursing workers, the words “psychological”, “family”, “health”, and “practice” were noted. The tree demonstrated that the primary strategies employed by nursing workers to cope with stress were family and psychological support, sought through personal resources. Despite the negative repercussions of the pandemic, the professionals identified positive aspects such as the effort to maintain positive thoughts, valuing workers in the field, overcoming fears, adopting healthy and relaxing practices, seeking spiritual support, and psychological assistance. These are effective strategies that help mitigate negative situations in the worker’s life^(22)^.

Regarding the job market, some participants reported finding their first job during the pandemic period, aiding in professional advancement and overcoming unemployment. Participants also reported personal changes, contributing to healthier interpersonal relationships between workers and patients, generating personal satisfaction for both the caregiver and the cared-for. Thus, the pursuit and practice of empathy strengthen the relationship of respect, understanding, active listening, and compassion^(18)^.

### Study limitations

The study’s limitations include the inability to interview the entire nursing staff due to withdrawals or refusals to participate in the research and the fact that the study was conducted in only one hospital unit. The data collection period during the pandemic context may not provide a complete perspective of the stress factors experienced by nursing workers.

### Contributions to the Field

This study contributed to the understanding of the stressors experienced by nursing workers caring for people with COVID-19 in a public hospital in the Recôncavo region of Bahia. Additionally, the results from this research will aid in the development of care strategies for these workers, aimed at reducing high levels of stress, which are extremely harmful to health and professional performance. This, in turn, can help improve the quality of life and, consequently, the care provided to patients in the hospital context. Academically, this study has the potential to motivate further research in this area, thereby contributing to the production of scientific knowledge in undergraduate and graduate nursing programs.

## FINAL CONSIDERATIONS

Stress is a factor that causes serious harm to nursing workers, both personally and professionally, posing risks to their quality of life and psychosocial well-being. The hospital environment fosters the emergence of stress, leading to changes in the physical and mental health of these individuals-an issue that was intensified during the pandemic, making it a significant area for scientific investigation and interest. It is necessary to formulate guidelines and public policies aimed at the prevention and promotion of worker health, in order to implement changes in working conditions, minimize stress-generating sources, and make the work environment more pleasant. Additionally, there is an urgent need to promote a humanized health system, with psychological support not only for the user but also for the worker, who is increasingly affected psychologically by the intense care they provide to others. Therefore, improvements in management policies and effective institutional support for nursing workers are proposed. Only in this way will it be possible to promote the mental health of these professionals, improving their quality of life and job satisfaction.
