# Supplementary material for: Stress in nursing workers caring for people with COVID-19
Source: Rev Bras Enferm. 2024 Dec 13;77(5):e20230542. doi: 10.1590/0034-7167-2023-0542 (PMC11654226; doi:10.1590/0034-7167-2023-0542)
Supplement: Translation

# Translation: O estresse em trabalhadores de enfermagem na atenção às pessoas com COVID-19

**DOI:** 10.1590/0034-7167-2023-0542pt

**Authors:** Carla Barbosa de Menezes, Maria Lúcia Silva Servo

**Affiliations:** I Universidade Estadual de Feira de Santana. Feira de Santana, Bahia, Brasil

**Keywords:** Estresse Ocupacional, Política de Saúde do Trabalhador, Saúde Mental, Enfermeiros, COVID-19

## Abstract

**Objetivos:**

analisar o estresse sob a perspectiva dos trabalhadores de Enfermagem na atenção às pessoas com COVID-19 em um hospital público do recôncavo baiano.

**Métodos:**

trata-se de uma pesquisa qualitativa de caráter exploratório, realizada por meio de entrevista semiestruturada. Os dados foram analisados via nuvens de palavras, árvore de similitude e análise de conteúdo.

**Resultados:**

os trabalhadores de Enfermagem ficaram expostos ao estresse ao atender pacientes com COVID-19. Os estressores relatados no ambiente laboral foram: sobrecarga de trabalho, falta de planejamento, rapidez na realização das tarefas, fadiga, falta de participação na tomada de decisões, falta de apoio por parte da gestão, mudanças tecnológicas, excesso de responsabilidade sem preparação, conflitos interpessoais e desvalorização profissional.

**Considerações Finais:**

o contato com os estressores acarreta desgaste emocional e desmotivação, intensificados na pandemia da COVID-19.

## INTRODUÇÃO

O estresse em trabalhadores de saúde - em particular, nos profissionais de Enfermagem - é um tema que fomenta muitos debates e investigações. A Enfermagem é uma categoria exposta a elevados níveis de pressão e estresse, que podem desencadear transtornos mentais caracterizados por sintomas como ansiedade, dificuldade de memória e concentração, fadiga, insônia e estresse^(1)^.

O estresse ocupacional difere do estresse comum pelo fato de ter o trabalho como fator essencial para o seu desenvolvimento; esse tipo de estresse ocorre quando o trabalhador não consegue agir sobre os agentes causadores, rompendo a adaptação, persistindo os sintomas de estresse, e o organismo é exaurido^(2)^.

É sabido que o hospital é um ambiente gerador de estresse para os trabalhadores, especialmente os de Enfermagem, que vivenciam múltiplas exigências, tais como: lidar com dor, sofrimento, morte e perdas, exigência de grande habilidade em lidar com usuários dos serviços, entre outros - o que, consequentemente, faz com que os trabalhadores fiquem mais vulneráveis a sentimentos de conteúdo depressivo e de esgotamento emocional. Acrescem a esses fatos o relacionamento interpessoal, as condições desfavoráveis de trabalho e a baixa remuneração, propiciando o desenvolvimento do estresse^(3)^.

Baseado no relatório da Organização Mundial da Saúde (OMS), divulgado pelo Conselho Federal de Enfermagem (COFEN)^(4)^, os fatores estressantes relacionados à atuação do trabalhador de Enfermagem tendem a se exacerbar diante de um cenário de calamidade pública global, como foi o caso da pandemia da COVID-19, que se instalou nos anos 2020, 2021 e 2022. Infelizmente, foram contabilizados 19.026 trabalhadores de Enfermagem confirmados com COVID-19, dentre os quais 833 vieram a óbito.

Estudos demonstraram que o número elevado de mortes e longas jornadas de trabalho sem estrutura adequada devido à COVID-19 em trabalhadores de Enfermagem e outros profissionais da área da saúde geraram estresse físico e emocional, depressão e insônia^(5-7)^. Diante disso, é importante desenvolver trabalhos que propiciem a elaboração de intervenções e medidas protetivas de cuidado para com os trabalhadores de Enfermagem.

## OBJETIVOS

Analisar o estresse em trabalhadores de Enfermagem, na atenção às pessoas com COVID-19, em um hospital público do recôncavo baiano.

## MÉTODOS

### Aspectos éticos

O projeto foi submetido e aprovado pelo Comitê de Ética em Pesquisa com Seres Humanos da Universidade Estadual de Feira de Santana (UEFS). O Termo de Consentimento Livre e Esclarecido (TCLE) foi assinado pelos participantes após serem informados quanto aos objetivos, riscos e benefícios da pesquisa. Os participantes foram identificados por meio das siglas de Entrevistado (ENT) e as numerações correspondentes à ordem de entrevista. Foi garantido ao participante da pesquisa o direito de não responder a qualquer questão, sem necessidade de explicação ou justificativa para tal, podendo também desistir da pesquisa a qualquer momento e sem nenhum prejuízo.

### Tipo de Estudo

Trata-se de um estudo descritivo de caráter qualitativo, realizado por meio de entrevista semiestruturada, acompanhada de um roteiro e construída em consonância com o instrumento *Consolidated Criteria for Reporting Qualitative Research* (COREQ)^(8)^. Este manuscrito é fruto da dissertação de mestrado apresentada ao Programa de Pós-Graduação em Saúde Coletiva da UEFS.

### Cenário de Estudo

O estudo ocorreu no Hospital Municipal Nossa Senhora da Natividade, localizado no município de Santo Amaro, no estado da Bahia. Os entrevistados são trabalhadores de Enfermagem que atuaram na atenção às pessoas com COVID-19, em pleno exercício profissional e inseridos no Cadastro Nacional de Estabelecimento de Saúde (CNES).

A instituição do estudo realiza atendimento de urgência e emergência; urologia, cardiologia, monitorização ambulatorial de pressão arterial e eletrocardiograma; neurologia com eletroencefalograma, entre outros. Todos os serviços são prestados para atender à demanda local e de municípios circunvizinhos. O hospital atende em média 250 pacientes/dia, possui 35 leitos para atendimento de urgência, emergência, adulto e infantil, clínica médica e internamento pediátrico. O local foi utilizado para o atendimento de pacientes diagnosticados com COVID-19 durante a pandemia, por um período de três anos, ofertando 15 leitos destinados à assistência a essas pessoas.

### Fonte de Dados

Para o acesso aos participantes do estudo, foi solicitada uma autorização da Secretaria Municipal de Saúde de Santo Amaro para a realização da pesquisa, bem como informações para contato, como telefone e e-mail dos trabalhadores vinculados à unidade.

Em um primeiro momento, 20 participantes, entre enfermeiros e técnicos de Enfermagem, foram abordados para a entrevista. Dentre eles, três desistiram alegando timidez e um demonstrou não ter interesse. Assim, totalizaram 16 participantes submetidos à entrevista por meio de gravação de áudio, com auxílio do celular para a realização das transcrições.

Os critérios de exclusão estabelecidos foram: trabalhadores de Enfermagem de licença médica ou licença-maternidade, de férias e/ou que não desejaram participar do estudo. No Quadro 1, foram caracterizados e descritos todos os participantes do estudo.

**Quadro 1:** Caracterização dos trabalhadores de Enfermagem que atuaram na atenção às pessoas com COVID-19 no Hospital do Recôncavo Baiano, em Santo Amaro, Bahia, Brasil, 2023

Nº	Idade	Sexo	Formação Profissional	Tempo de Formação	Tempo de Atuação	Tempo de Serviço na Instituição	Jornada de Trabalho	Outro Vínculo
**1**	27	Feminino	Técnico de Enfermagem	4 anos	4 anos	4 anos	36hrs/ semanal	Não
**2**	29	Feminino	Técnico de Enfermagem	4 anos	4 anos	4 anos	36hrs/ semanal	Não
**3**	25	Feminino	Técnico de Enfermagem	6 anos	5 anos	2 anos	36hrs/ semanal	Sim
**4**	36	Feminino	Técnico de Enfermagem	3 anos	3 anos	2 anos	36hrs/ semanal	Não
**5**	42	Feminino	Enfermeira	5 anos	5 anos	2 anos	36hrs/ semanal	Sim
**6**	43	Feminino	Enfermeira	4 anos	4 anos	4 anos	36hrs/ semanal	Sim
**7**	35	Feminino	Enfermeira	8 anos	4 anos	1 ano	36hrs/ semanal	Não
**8**	35	Feminino	Enfermeira	13 anos	12 anos	2 anos	36hrs/ semanal	Sim
**9**	54	Masculino	Enfermeira	4 anos	3 anos	2 anos	36hrs/ semanal	Não
**10**	36	Feminino	Enfermeira	7 anos	3 anos	3 anos	36hrs/ semanal	Não
**11**	34	Masculino	Enfermeira	3 anos	2 anos	2 anos	36hrs/ semanal	Não
**12**	36	Feminino	Enfermeira	3 anos	2 anos	2 anos	36hrs/ semanal	Não
**13**	24	Feminino	Técnico de Enfermagem	2 anos	2 anos	2 anos	36hrs/ semanal	Sim
**14**	50	Feminino	Técnico de Enfermagem	9 anos	6 anos	3 anos	36hrs/ semanal	Não
**15**	37	Feminino	Enfermeira	7 anos	7 anos	4 anos	36hrs/ semanal	Sim
**16**	46	Feminino	Técnico de Enfermagem	8 anos	8 anos	8 anos	36hrs/ semanal	Não

### Coleta e organização dos dados

A entrevista semiestruturada foi aplicada no período de dezembro de 2022 a fevereiro de 2023, segmentada em duas partes: a primeira, refere-se à caracterização dos participantes, e a segunda, ao estresse em trabalhadores de Enfermagem. As entrevistas foram realizadas pela pesquisadora principal, de forma individual, com agendamento prévio de data, horário e local, de acordo a disponibilidade do participante.

As questões norteadoras da entrevista foram:

1. O que você entende por estresse?
2. Descreva como foi o trabalho durante a pandemia?
3. Vivenciou alguma situação de estresse no seu cotidiano de atenção a pessoas com COVID - 19? Se sim, como você enfrentou essas situações?

A entrevista com o participante durava em torno de 20 a 30 minutos e, ao final, os participantes tiveram a oportunidade de ouvir as gravações, com o objetivo de realizar correções das respostas fornecidas, caso fosse pertinente, e para consentirem ou não com sua participação no projeto. O término das entrevistas ocorreu quando as concepções, explicações e sentidos atribuídos pelos participantes não mais contribuíram para a reflexão teórica e se tornaram repetitivas. Ressalta-se que, após realizar as transcrições, elas foram enviadas aos participantes para validação.

Concluída a coleta, foi realizado o download dos dados obtidos durante as gravações e o TCLE, e enviados para o e-mail pessoal da pesquisadora responsável. Posteriormente, os arquivos foram armazenados em um pen drive, para que todos os dados obtidos pudessem ser deletados do celular.

### Análise dos Dados

A caracterização dos participantes do estudo foi inserida no programa *Microsoft Word* ^®^. Além disso, os resultados das respostas referentes ao tema central dessa pesquisa foram organizados por meio das seguintes ferramentas:

- **Análise da Similitude:** o corpus textual foi analisado por meio do *software* IRAMUTEQ (*Interface de R pour les Analyses Multidimensionnelles de Textes et de Questionnaires*), versão *alpha* 2^(9)^. Esse é um software gratuito que funciona como uma interface de R, indicado para o gerenciamento e tratamento estatístico de textos de entrevistas e questionários abertos; a transcrição foi realizada no *software Apache OpenOffice* ^®^ para evitar erros relativos à codificação.
- **Nuvem de palavras:** Essa ferramenta organiza a entrevista de forma que, quanto maior for o tamanho da fonte e mais centralizada estiver a palavra na nuvem, maior é a representatividade do termo no corpus em análise. O contrário também ocorre: quanto mais afastada e menor seu tamanho, menor o seu grau de evocação^(10)^.
- **Análise de conteúdo:** Os dados da entrevista semiestruturada foram analisados e interpretados com base no Método de Análise de Conteúdo de Bardin^(11)^. Trata-se de uma categoria de métodos de análise das conversações que permite a dedução de conhecimentos relativos às condições de produção das mensagens, por procedimentos sistemáticos e objetivos de descrição do conteúdo. Além disso, esse método possibilita percorrer e analisar aquilo que vai além dos conteúdos manifestados.

## RESULTADOS

A partir dos dados obtidos, foram apreendidas as seguintes categorias: significado e vivência de estresse no olhar de trabalhadores de Enfermagem da área hospitalar que atuaram no período pandêmico da COVID-19 (Figura 1); e o estresse no cotidiano hospitalar dos trabalhadores de Enfermagem atuantes na atenção a pessoas com COVID-19 (Figura 2).

**Figura 1:** Significado e vivência de estresse por trabalhadores de Enfermagem da área hospitalar durante a pandemia da COVID-19

**Figura 2:** Estratégias de enfrentamento utilizadas por trabalhadores de Enfermagem da área hospitalar no período pandêmico da COVID-19

### Categoria 1 - Significado e vivência de estresse por trabalhadores de Enfermagem da área hospitalar durante a pandemia da COVID-19

O significado e a vivência de estresse perpassam pela compreensão que os trabalhadores de Enfermagem têm do estresse como uma ocorrência conflituosa, que afeta o equilíbrio emocional, gerando reações físicas e mentais, diante das situações vivenciadas por eles:

[...] *Situações que nos tira o equilíbrio emocional devido a situações negativas que acontecem na nossa vida* [...]. (Ent. dois)

[...] *tudo aquilo que nos tira da zona de conforto* [...] *muda o nosso cotidiano, desestabiliza a nossa saúde mental e gera desconforto no nosso cotidiano* [...]. (Ent. cinco)

Foi possível identificar a relação entre o conceito do estresse e os fatores externos decorrentes do ambiente laboral, bem como os sintomas causados com o excesso de estresse:

[...] *Estresse causa uma demanda excessiva que muitas vezes não conseguimos dar conta, é quando ocorre uma cobrança excessiva uma pressão, esses fatores são desencadeadores do estresse* [...]*. Saber que está sendo cobrado, pressionado* [...]*. Além de lidar com limitação e pouca condição para atender a alta demanda* [...]. (Ent. nove)

[...] *Estresse é a ocorrência de circunstâncias súbitas ou ameaçadoras, que gera um estado de alerta* [...]*. Situações que causam excitação, irritabilidade e medo* [...]. (Ent. oito)

Em relação aos estressores que afetam a vida dos trabalhadores, foi relatado que:

[...] *tentar resolver problemas e solucionar coisas que não estavam ao alcance, lidar com a ausência da família, lidar com as famílias querendo acesso aos pacientes e não ser possível* [...] *fato de ser algo novo e desconhecido,* [...] *a falta de informação,* [...] *falta de equipamentos* [...] *um ambiente de trabalho muito hostil* [...] *lidava com situações de estresse todos os dias na vivência da assistência* [...]. (Ent. 13)

A sobrecarga de trabalho causada pela jornada excessiva interfere negativamente na qualidade de vida, como foi constatado por um dos entrevistados:

[...] *sobrecarga de trabalho, afastamento de profissionais* [...] *e insuficiência de equipamentos de proteção individual,* [...] *risco de contaminação pelo COVID-19* [...] *uso prolongado de máscara, fadiga crônica, discriminação, violência física e verbal,* [...] *as jornadas exaustivas elevou o nível de estresse e insatisfação comprometendo assim a qualidade de serviço do trabalho, frustração de não conseguir salvar a vida apesar de todo o meu esforço* [...] *a dor da perder sem ao menos se despedir* [...]. (Ent. oito)

### Categoria 2 - O estresse no cotidiano hospitalar dos trabalhadores de Enfermagem atuantes na atenção a pessoas com COVID-19

Os trabalhadores de Enfermagem vivenciaram situações de discriminação e isolamento por parte de amigos, familiares e conhecidos - por trabalharem na linha de frente, acabavam representando uma fonte de contaminação para a sociedade. É o que o entrevistado a seguir ratifica:

[...] *vivenciei o preconceito por termos contato direto com os pacientes portadores do vírus e ser da área da saúde, as pessoas e os próprios parentes achavam, que éramos um transmissor da doença* [...]. (Ent.um)

Quanto ao estresse no cotidiano dos trabalhadores de Enfermagem na atenção a pessoas com COVID-19 em hospital, foi relatado que:

[...] *existia um medo de contrair a doença e transmitir para as pessoas ao nosso redor.* (Ent. dois)

[...] *lidar com o medo do desconhecido* [...]. (Ent. 14)

Os trabalhadores de Enfermagem compreendem o medo exacerbado como um sentimento presente naquele momento, especialmente em decorrência do medo da morte, da transmissão e da contaminação em meio ao caos instalado. Isso fica evidente com o seguinte depoimento:

[...] *fiquei com bastante ansiedade, estresse e medo* [...] *medo de morrer* [...]. (Ent. quatro)

A Pandemia da COVID-19 trouxe grandes repercussões na saúde mental dos trabalhadores de Enfermagem. Essa questão foi exposta na entrevista abaixo:

[...] *incertezas* [...] *além do excesso de informações na mídia a politização da saúde aumentava cada vez mais o meu estresse a minha insegurança, com meus familiares e amigos causando um afastamento com medidas de isolamento, perda do sono, irritabilidade, choros frequentes, incapacidade de relaxar, dificuldade de concentração, pensamentos lentos são os aspectos que mais me evidenciou* [...]. (Ent. oito)

Diante dos relatos, nota-se que há sensação de insegurança, juntamente com a sensação de impotência:

[...] *sentia muita ansiedade, estresse, não conseguia dormir, ficava com insônia, pensando, não conseguia comer, na época perdi peso, sentia muito insegurança, tristeza, chorava muito e chegava em casa todas as vezes que eu voltava para casa eu chorava demais me sentindo impotente* [...]. (Ent. 12)

[...] *Impossibilidades de muitas vezes não poder dar suporte adequado ao paciente no pedido de atenção* [...] *a carência dos pacientes pela falta de acompanhante, mas a sobrecarga impossibilitava tentar suprir, déficit de equipamento para a quantidade de pacientes que necessitava, impossibilidade de uma palavra de conforto no momento de solidão, vivi momentos de tristeza, em ver o sofrimento dos pacientes sem o familiar por perto, isolado mas todo tempo possível a equipe estava dando atenção a eles* [...]. (Ent. seis)

Houve situações negativas na saúde mental do trabalhador, demonstrada por meio dos vários sinais e sintomas:

[...] *comecei a ter insônia, irritabilidade, choro frequentes, não conseguia relaxar e dificuldade de concentração, perda de satisfação na carreira* [...]. (Ent. seis)

[...] *foi o déficit de material, equipamento, déficit de muita coisa devido a demanda faltava os materiais, máscara, luva não só na cidade onde a gente está falando agora, mas em todo o país em todo mundo estava faltando equipamentos, materiais para prestar assistência ao paciente com COVID-19* [...]. (Ent. cinco)

Nesse sentido, as estratégias de enfrentamento utilizados pelos trabalhadores de Enfermagem, frente às situações do estresse no cotidiano hospitalar foram as seguintes:

[...] *Chamar por Deus para nos dar controle emocional para agir na assistência da melhor forma* [...]. (Ent. 14)

[...] *Eu enfrentei essa situação sempre com pensamento positivo, isso me ajudou a não me desesperar ou até mesmo entrar em depressão como muitos outros profissionais* [...] *infelizmente”.* (Ent. dois)

[...] *equilíbrio emocional* [...]*, um verdadeiro preparo psicológico* [...]. (Ent. nove)

[...] *tive que controlar o meu emocional* [...] *eu tenho dificuldade da ajuda psicológica a distância* [...]. *Não funcionou, eu tentei, mas não funcionou e para mim eu tive que realmente tentar controlar o meu emocional* [...]. (Ent. 10)

Apesar do período pandêmico, a oportunidade de conquistar o primeiro trabalho foi algo positivo para alguns dos entrevistados:

[...] *contratação da mão-de-obra imediata, que a partir daí abriram-se as portas do emprego para os profissionais principalmente aqueles que estavam desempregados e não tinham experiência, estavam em busca de uma oportunidade no mercado de trabalho* [...]. (Ent. um)

[...] *oportunidade de novos empregos, porque esse foi o meu primeiro emprego, e eu estava procurando antes do COVID-19 surgir* [...]. (Ent. 10)

[...] *que eu era recém-formada abriu uma porta de emprego* [...]. (Ent. 12)

[...] *Possibilidade de emprego principalmente que estava desempregada* [...]. (Ent. 14)

Os participantes também relataram pontos referentes às relações empáticas vivenciadas por eles durante o período da pandemia:

[...] *não cruzar os braços e fazer a diferença na vida de cada pessoa que passava pelas nossas mãos sempre dando o melhor, consolei muitas famílias e chorei também com as mesmas pela perda de seus entes queridos, mas também vi muitos pacientes recuperados e voltando para o seio dos seus familiares* [...]. (Ent. dois)

[...] *a mudança de um olhar sobre a vida, sobre o meu conceito de assistência, sobre o meu conceito de humanidade, de tratamento e de respeito à vida do outro* [...]. (Ent. 13)

[...] *sempre tendo a consciência tranquila de que estava fazendo o melhor, a sensação de dever cumprido e poder ajudar as pessoas era gratificante, outra possibilidade é o avanço enquanto ser humano foi uma lição para todos nós, conseguir evoluir muito enquanto pessoa, enquanto profissional, porque eu acabei tendo uma visão diferente, um crescimento enquanto ser humano, enquanto espírito* [...]. (Ent. 15)

Na entrevista, também foram destacadas algumas situações positivas no cotidiano do indivíduo. Essas situações levaram a mudanças na rotina diária e se tornaram hábito na vida do trabalhador:

[...] *um grande legado que a ciência avançou, se descobriu novas tecnologia, equipamentos que antes eram usados em menor escala hoje está sendo mais usados, a exemplo do oxímetro, em relação a higiene foi algo que a população passou a desenvolver hábito de lavar as mãos constantemente, manter uma higiene pessoal melhor com mais assiduidade, perspectivas para esse momento foi que as pessoas evoluíram no aspecto da higiene, no aspecto do autocuidado* [...]. (Ent. dois)

## DISCUSSÃO

O momento pandêmico foi relevante para analisar o estresse vivenciado pelos trabalhadores de Enfermagem, pois havia o medo exacerbado ao lidar com a iminência de morte, medo de se contaminar e transmitir a doença - principalmente a familiares. Diante disso, o desenvolvimento de sentimentos de incerteza, insegurança, impotência, irritabilidade e insônia foi destacado pelos trabalhadores de Enfermagem. Frente a esses fatores, houve estratégias de enfrentamento, como o aprofundamento na religião para fortalecer a fé e a busca por apoio psicológico por meio de recursos próprios, sem ajuda institucional. De maneira positiva, houve um aumento na procura por trabalhadores de Enfermagem e o fortalecimento das relações empáticas, ocasionando mudanças significativas no ambiente laboral.

Os participantes englobados nessa pesquisa eram, em sua maioria, do sexo feminino^(12)^, na faixa etária entre 24 e 54 anos, quadro compatível com os achados de Appel, Carvalho e Santos^(13)^, onde a equipe de Enfermagem era composta predominantemente por mulheres (88,5%) na faixa entre 23 e 54 anos, com maior vulnerabilidade para desenvolver estresse, ansiedade e depressão. A prevalência de sintomas de estresse em mulheres pode ser justificada pela sobrecarga ocasionada pela associação do trabalho formal e o doméstico, incerteza em relação a questões socioeconômicas-emocionais, risco de desemprego, atividades de home office que passaram a compatibilizar atividades familiares e profissionais, aumento de ocorrências de violência doméstica nesse período de isolamento e o medo de contaminar seus familiares com a COVID-19^(14-16)^.

Observou-se uma variação de dois a quatro anos atuando neste estabelecimento de saúde, e um total de oito participantes iniciaram o serviço no período pandêmico. Sabe-se que, quanto menor a experiência, maior a predisposição ao estresse relacionado ao trabalho, por apresentarem maior dificuldade em lidar com os estressores encontrados, o que acarreta a escassez de trabalhadores na Enfermagem. Esta condição está em consonância com a pesquisa, uma vez que os trabalhadores possuíam curto tempo de atuação profissional. Profissionais mais experientes acabam tendo melhores condições de enfrentamento diante das situações estressoras^(14)^.

Os trabalhadores de Enfermagem da rede hospitalar entrevistados possuíam, em média, uma carga horária de 36 horas semanais, em caráter de plantões diurnos e noturnos, e a grande maioria tinha 12 ou mais anos de experiência profissional. Sabe-se que, quanto maior o tempo de exposição às circunstâncias que geram desgaste físico e cognitivo no ser humano, maior a vulnerabilidade na saúde psicológica do trabalhador^(16)^.

A Enfermagem apresenta a maior força de trabalho da saúde no mundo e foi a classe que apresentou mais medo de adquirir o vírus devido ao risco de contágio. Essa foi a categoria que mais se contaminou, com evolução ao óbito, no período pandêmico da COVID-19^(17)^. Tendo isso como premissa, a palavra “estresse”, na análise de similitude, foi a mais centralizada, encontrando-se interligada às palavras “vivenciar” e “excessivo”, o que sugere a relação do estresse com a ocorrência de situações que afetam a saúde mental vivenciadas em excesso.

A palavra “situação” está acompanhada das palavras “COVID-19”, “cotidiano” e “mudança”, demonstrando que as alterações sofridas pelos trabalhadores de Enfermagem no seu cotidiano hospitalar afetam a saúde mental no período da COVID-19 e desencadeiam o estresse. Vivências relacionadas ao trabalho no período da COVID-19 são prejudiciais, como as particularidades das organizações do ambiente de trabalho, a sobrecarga de trabalho causada pela jornada excessiva, as demandas psíquicas constantes e elevadas, questões habituais de desempenho, número escasso de trabalhadores e alternância nas jornadas de trabalho^(18)^. Desta forma, considera-se que o cenário ocasionado pela pandemia da COVID-19 trouxe inúmeras mudanças no cotidiano hospitalar, o que gerou desgaste físico e psicológico desses trabalhadores, desencadeando, consequentemente, o estresse.

As constantes situações de estresse vivenciadas por trabalhadores de Enfermagem foram potencializadas no período pandêmico da COVID-19, pois foi necessário lidar com a morte, escassez de EPIs e elevados números de pacientes com alto poder de transmissibilidade viral^(19)^. Somado a isso, existia a necessidade de desenvolver um atendimento preciso e cauteloso, tanto nos procedimentos técnicos quanto no autocuidado, buscando minimizar os riscos de contaminação. Esses fatores acarretaram adoecimento físico e mental, podendo afastar os trabalhadores de Enfermagem das suas tarefas laborais^(20)^.

O medo é necessário para a sobrevivência, pois envolve processos biológicos que contribuem para a defesa do organismo na resposta a acontecimentos ameaçadores. Quando exacerbado, como no relato dos participantes deste estudo, torna-se danoso, contribuindo para o desenvolvimento do estresse e outros transtornos psiquiátricos, como o nervosismo, incapacidade de relaxar, insônia, sonolência diurna, incerteza e insegurança frente às informações disponibilizadas pela mídia com relação às condutas de tratamento, atreladas à incerteza e à imprevisibilidade do período pandêmico da COVID-19, desencadearam uma predisposição para o surgimento do estresse entre trabalhadores de Enfermagem^(21-24)^. A incerteza influencia de forma negativa no comportamento e no bem-estar geral dos trabalhadores de Enfermagem e, consequentemente, interfere na sustentação da qualidade dos cuidados em saúde destinados à população^(25)^. Ademais, o sentimento de impotência aflora quando esses trabalhadores não são capazes de modificar a situação crítica do paciente^(18,26)^.

### As estratégias de enfrentamento utilizada pelos trabalhadores de Enfermagem

Notou-se na árvore de similitude, referente às estratégias de enfrentamento utilizadas pelos trabalhadores de Enfermagem, as palavras: psicológico, familiar, saúde e prática. A árvore demonstrou que as principais estratégias utilizadas pelos trabalhadores de Enfermagem para enfrentar o estresse foram o apoio familiar e psicológico - sendo este buscado por meio de recursos próprios. Além das repercussões negativas da pandemia, os profissionais conseguiram identificar aspectos positivos, como a busca em manter pensamentos positivos, valorizar o trabalhador da área, abdicar dos medos, aderir a práticas saudáveis e de relaxamento, buscar apoio espiritual e suporte psicológico. Essas são estratégias efetivas que auxiliam para diminuir situações negativas na vida do trabalhador^(22)^.

No que se refere ao mercado de trabalho, alguns dos participantes relataram o surgimento do primeiro emprego no período pandêmico, ajudando na ascensão profissional e na superação do desemprego. Os participantes também relataram a mudança do indivíduo em sua particularidade, contribuindo para uma relação interpessoal mais saudável entre os trabalhadores e pacientes, gerando satisfação pessoal tanto em quem cuida como em quem é cuidado. Assim, a busca e o exercício da empatia fortalecem a relação de respeito, compreensão, escuta ativa e compaixão^(18)^.

### Limitações do estudo

As limitações do estudo incluem a impossibilidade de entrevistar todo o corpo de profissionais de Enfermagem devido à desistência ou recusa de participar da pesquisa, e o fato da pesquisa ter sido realizada somente em uma unidade hospitalar. O período de coleta dos dados no contexto pandêmico pode não apresentar uma perspectiva completa dos fatores estressantes gerados nos trabalhadores de Enfermagem.

### Contribuições para a Área

Este estudo contribuiu para o conhecimento das situações estressoras vivenciadas por trabalhadores de Enfermagem que atuam na atenção a pessoas com COVID-19 em um hospital público do recôncavo baiano. Ademais, os resultados advindos desta pesquisa propiciarão a elaboração de estratégias de cuidado para com os trabalhadores que se dedicam a esse ofício, de forma a reduzir altos níveis de estresse, que são extremamente prejudiciais à saúde e ao desempenho profissional. Desta forma, será possível colaborar com a melhoria da qualidade de vida e, consequentemente, da assistência prestada aos pacientes no contexto hospitalar. No âmbito acadêmico, este estudo tem a possibilidade de motivar a realização de novas pesquisas na área, a fim de contribuir com a produção de conhecimento científico nos cursos de graduação e de pós-graduação em Enfermagem.

## CONSIDERAÇÕES FINAIS

O estresse é um fator que acarreta sérios prejuízos ao trabalhador de Enfermagem, tanto no âmbito pessoal quanto laboral, gerando riscos à qualidade de vida e ao bem-estar psicossocial. O ambiente hospitalar propicia o surgimento do estresse, ocasionando alterações na saúde física e mental desses indivíduos - situação que foi intensificada diante do enfrentamento no período de pandemia, tornando-se um fator importante de investigação e interesse científico.

É necessária a formulação de diretrizes e políticas públicas no âmbito de prevenção e promoção à saúde do trabalhador, de modo a articular mudanças nas condições de trabalho, minimizando fontes geradoras de estresse e tornando o ambiente laboral mais prazeroso. Além disso, é evidente a urgência de promover um sistema de saúde humanizado, com apoio psicológico não apenas para o usuário, mas também para o trabalhador, que está cada vez mais afetado psicologicamente, trabalhando de maneira intensa no cuidado ao ser humano. Propõe-se, assim, uma melhoria nas políticas de gestão e apoio institucional efetivo para os trabalhadores de Enfermagem. Somente desta forma será possível promover a saúde mental desses profissionais, melhorando sua qualidade de vida e sua satisfação no trabalho.
